# Three distinct conductance states in polycyclic aromatic hydrocarbon derivatives

**DOI:** 10.1098/rsos.231734

**Published:** 2024-06-12

**Authors:** Ali K. Ismael, Taha Abdel Mohaymen Taha, Alaa Al-Jobory

**Affiliations:** ^1^ Department of Physics, Lancaster University, Lancaster LA1 4YB, UK; ^2^ Department of Physics, College of Education for Pure Science, Tikrit University, Tikrit, Iraq; ^3^ Physics and Engineering Mathematics Department, Faculty of Electronic Engineering, Menoufia University, Menouf 32952, Egypt; ^4^ Department of Physics, College of Science, University of Anbar, Anbar, Iraq

**Keywords:** distinct, conductances, states, polycyclic aromatic hydrocarbon, derivatives, density functional theory

## Abstract

Tight-binding model (TBM) and density functional theory (DFT) calculations were employed. Both simulations have demonstrated that the electrical conductance for eight polycyclic aromatic hydrocarbons (PAHs) can be modulated by varying the number of aromatic rings (NAR) within the aromatic derivatives. TBM simulations reveal three distinct conductance states: low, medium and high for the studied PAH derivatives. The three distinct conductance states suggested by TBM are supported by DFT transmission curves, where the low conductance evidenced by *T*(*E*) = 0, for benzene, naphthalene, pyrene and anthracene. While azulene and anthanthrene exhibit a medium conductance as *T*(*E*) = 1, and tetracene and dibenzocoronene possess a high conductance with *T*(*E*) = 2. Low, medium and high values were elucidated according to the energy gap *E*_g_ and *E*_g_ gaps are strongly dependent on the NAR in the PAH derivatives. This study also suggests that any PAH molecules are a conductor if *E*_g_ < 0.20 eV. A linear relationship between the conductance and NAR (*G *∝ NAR) was found and conductance follows the order *G* (benzene, 1 NAR) < *G* (anthanthrene, 4 NAR) < *G* (dibenzocoronene, 9 NAR). The proposed study suggests a relevant step towards the practical application of molecular electronics and future device application.

## 1. Introduction

π−π stacking is a fundamental feature owing to its several controls of substantial physical properties within condensed matter physics such as phonon transport, charge transfer and energy transfer [1]. π-stacks including intra- and intermolecular are deemed to play structural and electronic roles in molecular recognition, self-assembly [2], organic electronics, DNA molecules [3,4] and protein folding [5] and π-stacks have been attracting notable interest [6–11]. π-systems were employed as building blocks to construct various electronic devices involving electronic tunnelling through individual π-conjugated molecules, examined by scanning tunnelling microscopy [12–14]. It has been proved that single π-conjugated molecules including hexa-peri-hexabenzocoronenes [15] and coronenes [16] placed on metallic electrodes present a rectification feature. π-conjugated molecules and π-stacks need to be attached to metallic electrodes so that they can be used in electronic device applications. Within this scope, the charge transfer via π-stacks in metal junctions has been explored, as well as further rectification properties and efficient charge transport properties being proven [17–20]. In 2020, Melikova and his group [21] considered aromatic compounds without a dipole moment, which makes it possible to reveal the contribution of quadrupole–quadrupole interactions to the π-stacking energy. These interactions are significant for heterodimers formed by arenes with more than two rings, with absolute values of the traceless quadrupole moment (Qzz) larger than 10 D Å. Furthermore, Singh *et al.* [22] showed that the perylene and naphthalene inclusion complexes G7CH and G1CH have the highest and lowest interaction energies, respectively. Furthermore, energy decomposition analysis indicated that the dispersion interaction term, DEdisp, significantly contributes to the host–guest interaction and is correlated with the existence of π–π van der Waals interaction.

Nevertheless, controlling the conductance does not come without difficulties as there is no direct method to control the stacking distribution in the π-stacks that affects the electronic properties. As an illustration, π-stacks of single molecules have been structured by employing guest aromatic molecules into host aromatic cages [23] or by covalently linking aromatics using strained paracyclophane backbones [13]. Nonetheless, a full control over the stacking distribution in the π-stacks has not been achieved because of the random molecular orientation [17–19,23–25]. The same work [16] of the π-stacks demonstrated that the molecular orientation of the π-stacks was not controlled on a gold surface. Other properties of polycyclic aromatic hydrocarbons (PAHs), involving phonon transport [26], charge transfer [27] and energy transfer [28], have also been explored.

**Figure 1.**
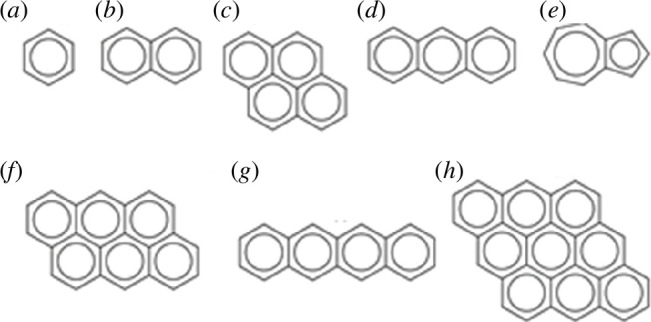
Schematic illustration of an oligoacene series including benzene (1NAR), naphthalene (2NAR), pyrene (4NAR), anthracene (3NAR), and azulene (2NAR) (*a–e*), and PAHs formed as clusters of anthracene molecules: anthanthrene (6NAR), tetracene (4R) and dibenzocoronene (9NAR) (*f–h*). Note: NAR refers to the number of aromatic rings in each PAH.

In the present work, we investigate the electronic properties and electric transport of an oligoacene series including benzene, naphthalene, azulene, anthracene, pyrene, tetracene and PAHs, formed as clusters of anthracene molecules as shown in figure 1. The major investigation here is dedicated to the electric transport using two different approaches: tight-binding model (TBM) and density functional theory (DFT). Additionally, further investigations were dedicated to the electronic structure properties including optimization, energy levels, degeneracy states, band structures, energy gap analyses and conducting differences. These parameters have a significant effect on the electric and thermoelectric transport in PAHs. By exploring the parameters mentioned above, it might be possible to answer whether aromaticity influences the electronic properties or not in PAHs.

## 2. Results and discussion

**Figure 2.**
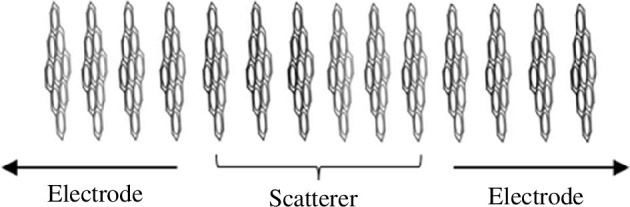
A crystalline structure of identical π-stacked PAH molecules (dibenzocoronene), divided into a scatterer connected to left and right electrodes.

In our previous work [29], we explored the electronic properties of the eight PAHs shown in figure 1. The current work focuses on the electrical conductance of these PAHs. To calculate the transmission coefficient *T*(*E*) through these PAHs, an infinite periodic structure of π-stacked molecules is required as illustrated in figure 2.

We began our theory by constructing a TBM, Hamiltonian from parameters for the hopping elements obtained from the band structures given by the DFT simulations. TBM is an analytical method that describes a structure based on the wave function of an electron as a linear combination of atomic orbitals of localized states. This model investigates electronic transport properties through the Hamiltonian of a finite set of atomic orbitals. The TBM approach assumes that electrons in a molecule form tightly bound interactions with only their nearest neighbouring sites.

TBM calculations demonstrate three distinct types of conductances: low, medium and high for the studied PAH derivatives. Electronic supplementary material, figure S4, shows that the conductance is low (*T*(*E*) = 0) for four PAHs as follows: benzene, naphthalene, pyrene and anthracene. While azulene and anthanthrene exhibit a medium conductance (*T*(*E*) = 1), and tetracene and dibenzocoronene possess a high conductance (*T*(*E*) = 2).

For further investigation and to support the TBM findings, DFT simulations on the same PAH derivatives were completed. The optimum geometries of isolated PAH derivatives were obtained by relaxing the molecules until all forces on the atoms were <0.01 eV Å^−1^ (electronic supplementary material, figure S1). We used a double-zeta plus polarization orbital basis set, norm-conserving pseudopotentials, the local density approximation (LDA) exchange-correlation functional, and an energy cutoff of 250 rydbergs to define the real space grid. We also computed the results using the generalized gradient approximation (GGA) and found that the resulting transmission functions were comparable [30–33] to those obtained using LDA. To simulate the likely contact configuration during a break-junction experiment, we used leads constructed from six layers of PAHs. After relaxing each molecular stack in different orientations, we calculated the electrical conductance using the Gollum quantum transport code [34].

**Figure 3.**
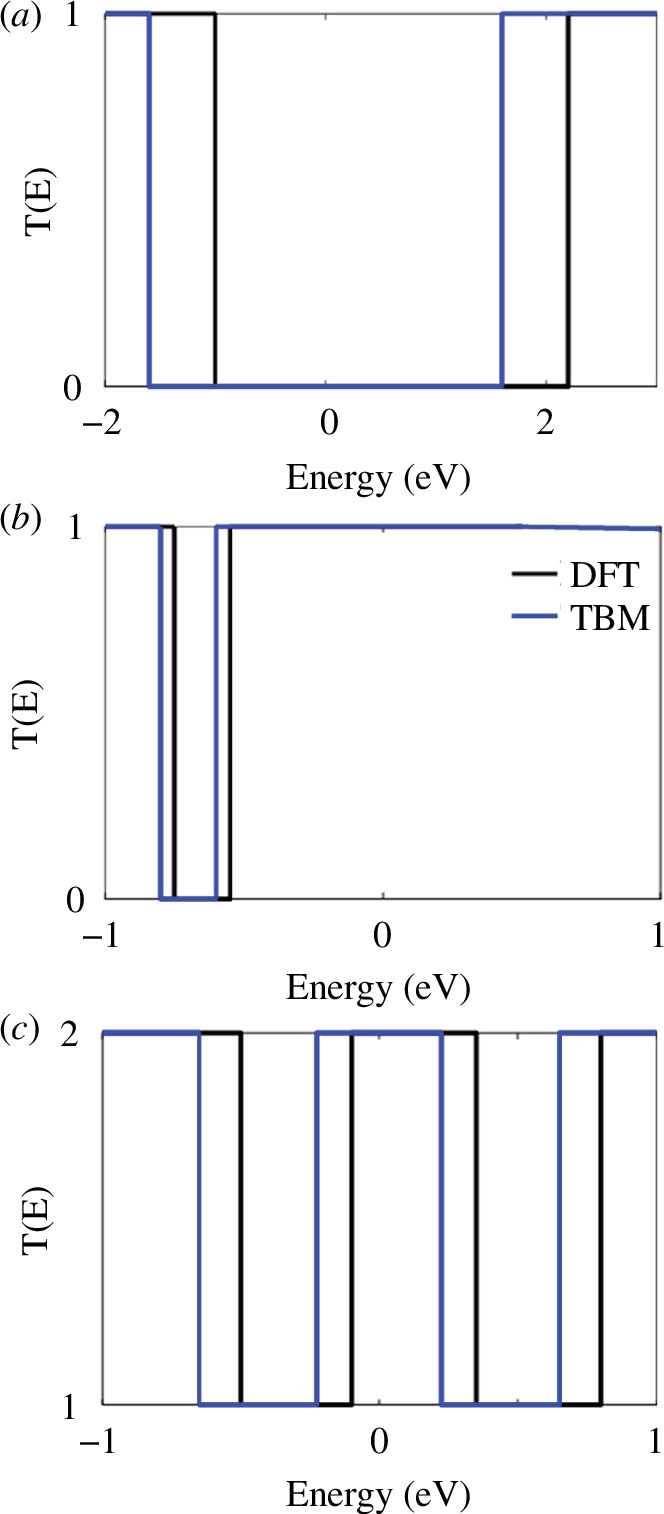
Calculated zero-bias transmission coefficient *T*(*E*), obtained from DFT (black curves) and the TBM (blue curves). (*a*) Transmission coefficient *T*(*E*) = 0 for benzene (low), (*b*) transmission coefficient *T*(*E*) = 1 for anthanthrene (medium) and (*c*) transmission coefficient *T*(*E*) = 2 for dibenzocoronene (high).

Figure 3 demonstrates the transmission coefficients for three PAH molecules. *T*(*E*) curves of these PAHs again exhibit the three distinct conductance states low, medium and high for benzene, azulene and dibenzocoronene (figure 3*a*–*c*, respectively). It should be noted that DFT results reproduce the TBM curves with a slight shift in the energy of electrons.

Figure 3 also manifests that TBM successfully captures the qualitative aspects of DFT simulations in connection with transport through PAH derivatives. This remains true on the assumption that the linkers solely consist of a single π-system. On the contrary, when the linkers contain more than one π-system, TBM requires to be improved in complexity by considering multiple orbital levels for each atomic site. This modification is fundamental to accurately account for the additional intricacies caused by multiple π-systems in the linkers. The three distinct conductance states of low, medium and high that are elucidated by both DFT and TBM can be easily explained by the band structure calculations for the studied PAH derivatives. Electronic supplementary material, figures S2 and S3, presents the band structure for each PAH molecule. Figure S2 determines the gap for the smallest PAH (benzene) to be large, approximately 3.25 eV.

**Figure 4.**
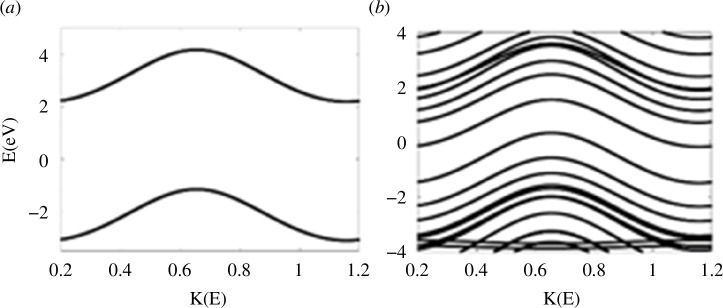
DFT band structures of single- and multi-ring molecules. Benzene 1R (*a*) and dibenzocoronene 9R (*b*). Note: other band structures are shown in electronic supplementary material, figures S2 and S3.

This large gap narrows with increasing number of aromatic rings (NAR) in PAH derivatives. The relationships between the energy gap ( $E_{g}$ ) and the NAR are as follows: $1R\;\left(benzene\right)\approx E_{g}=3.25\;eV$ , $2R\;\left(naphthalene\right)\approx E_{g}=1.51\;eV$ , $4NAR\;\left(pyrene\right)\approx E_{g}=0.78\;eV$ , $3R\;\left(anthracene\right)\approx E_{g}=0.42\;eV$ , $2NAR\;\left(azulene\right)\approx E_{g}=0.39\;eV$ , $6NAR\;\left(anthanthrene\right)\approx E_{g}=0.18\;eV$ , and the energy gap disappears ( $E_{g}\approx 0\;eV$ ) for both tetracene (4NAR) and dibenzocoronene (9NAR), as shown in table 1. Large gap and no gap of benzene and dibenzocoronene are shown in figure 4.

**Table 1.** Energy gap values for the studied molecules (PAHs), with their NAR.

PAH	NAR	*E*_*g*_ (eV)
benzene	1	3.25
naphthalene	2	1.51
pyrene	4	0.78
anthracene	4	0.42
azulene	2	0.39
anthanthrene	6	0.18
tetracene	4	0.0
dibenzocoronene	9	0.0

Band structure values suggest that the electrical conductance for broad bands to be low as illustrated in figure 3*a*, where *T*(*E*)= 0 for benzene. Medium conductance *T*(*E*) = 1 for small band gap (0.18 eV), such as anthanthrene (figure 3*b*), and high conductance *T*(*E*) = 2 for no gap including tetracene and dibenzocoronene as shown in figure 3*c*. For more detail, see band structure section and electronic supplementary material, figures S3 and S4.

The three distinct conductance states scenario in PAH derivatives is supported by the DFT energy gaps estimated using two different methods including GGA and B3LYP. GGA and B3LYP functions determine the energy gap to be 5 and 7 eV for benzene (GGA and B3LYP respectively), 2 and 3.2 eV for anthanthrene, while 1.1 and 2.7 for dibenzocoronene. The DFT simulations on the energy gap for aromatic molecular rings are supported by experimental measurement [35–39] as shown in electronic supplementary material, table S1. Despite its simplicity, TBM provides accurate results and this is why it has been chosen over DFT initially.

## 3. Conclusion

In conclusion, through rational TBM and DFT simulations we have demonstrated that the electrical conductance of eight PAHs can be modulated by varying the NAR within the aromatic derivatives. TBM simulations reveal three distinct conductance states: low, medium and high for the studied PAH derivatives. The three distinct conductance states of TBM are also supported by DFT transmission curves, where the low conductance is evidenced by *T*(*E*) = 0 for benzene, naphthalene, pyrene and anthracene. While azulene and anthanthrene exhibit a medium conductance *T*(*E*) = 1, and tetracene and dibenzocoronene possess a high conductance with *T*(*E*) = 2.

Band structure plots suggest an inverse correlation between the NAR and band gap size ( $E_{g}$ ). These simulations suggest that the band gaps follow the order $E_{g}\;\left(benzene\right)$ **>** $E_{g}\;(naphthalene)$ **>** $E_{g}\;\left(pyrene\right)$ **>** $E_{g}\;(anthracene)$ **>** $E_{g}\;\left(azulene\right)$ **>** $E_{g}\;(anthanthrene)$ **>** $E_{g}\;\left(tetracene\right)$ **>** $E_{g}\;(dibenzocoronene)$ . The same calculations were repeated, however, in the gas phase for the eight PAHs, and the same trend was obtained. This work sheds a light on new ideas for designing electronic devices based on using different size of PAHs, focusing on multi-ring derivatives, with potential practical applications.

## Data Availability

Supplementary data available online at [40].
